# Pd(II) and Pt(II) Trinuclear Chelates with Spermidine: Selective Anticancer Activity towards TNBC-Sensitive and -Resistant to Cisplatin

**DOI:** 10.3390/pharmaceutics15041205

**Published:** 2023-04-10

**Authors:** Martin Vojtek, Clara B. Martins, Raquel Ramos, Sara Gomes Duarte, Isabel M. P. L. V. O. Ferreira, Ana L. M. Batista de Carvalho, M. Paula M. Marques, Carmen Diniz

**Affiliations:** 1 LAQV/REQUIMTE, Laboratory of Pharmacology, Department of Drug Sciences, Faculty of Pharmacy, University of Porto, 4050-313 Porto, Portugal; 2 Molecular Physical-Chemistry R & D Unit, Department of Chemistry, University of Coimbra, 3004-535 Coimbra, Portugal; 3 LAQV/REQUIMTE, Laboratory of Bromatology and Hydrology, Department of Chemical Sciences, Faculty of Pharmacy, University of Porto, 4050-313 Porto, Portugal; 4 Department of Life Sciences, Faculty of Science and Technology, University of Coimbra, 3000-456 Coimbra, Portugal

**Keywords:** palladium, platinum, cisplatin, therapy, antiproliferative, cell viability, antineoplastic activity, cancer resistance

## Abstract

Triple-negative breast cancer (TNBC) is one of the most aggressive forms of breast cancer and constitutes 10–20% of all breast cancer cases. Even though platinum-based drugs such as cisplatin and carboplatin are effective in TNBC patients, their toxicity and development of cancer drug resistance often hamper their clinical use. Hence, novel drug entities with improved tolerability and selectivity profiles, as well as the ability to surpass resistance, are needed. The current study focuses on Pd(II) and Pt(II) trinuclear chelates with spermidine (Pd_3_Spd_2_ and Pt_3_Spd_2_) for evaluating their antineoplastic activity having been assessed towards (i) cisplatin-resistant TNBC cells (MDA-MB-231/R), (ii) cisplatin-sensitive TNBC cells (MDA-MB-231) and (iii) non-cancerous human breast cells (MCF-12A, to assess the cancer selectivity/selectivity index). Additionally, the complexes’ ability to overcome acquired resistance (resistance index) was determined. This study revealed that Pd_3_Spd_2_ activity greatly exceeds that displayed by its Pt analog. In addition, Pd_3_Spd_2_ evidenced a similar antiproliferative activity in both sensitive and resistant TNBC cells (IC_50_ values 4.65–8.99 µM and 9.24–13.34 µM, respectively), with a resistance index lower than 2.3. Moreover, this Pd compound showed a promising selectivity index ratio: >6.28 for MDA-MB-231 cells and >4.59 for MDA-MB-231/R cells. Altogether, the data presently gathered reveal Pd_3_Spd_2_ as a new, promising metal-based anticancer agent, which should be further explored for the treatment of TNBC and its cisplatin-resistant forms.

## 1. Introduction

**Figure 1:**
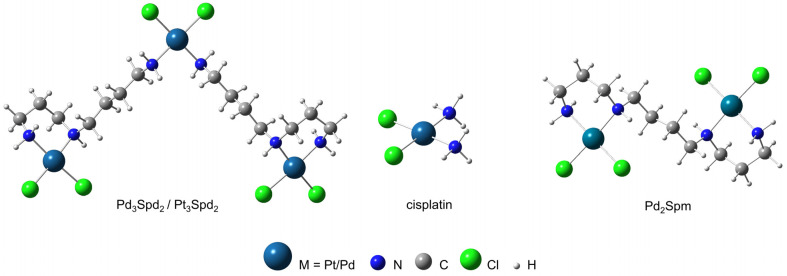
Structure of Pt(II)- and Pd(II)-based agents: Pd(II) or Pt(II) trinuclear chelates with spermidine, Pd_3_Spd_2_ or Pt_3_Spd_2_; conventional mononuclear Pt(II) drug—cisplatin and Pd(II) dinuclear chelates with spermine Pd_2_Spm.

Cancer resistance to drugs is driven by the reduction or even the lack of an effective response to drug-induced cancer growth inhibition. Resistance can occur in drug-naïve patients, also known as intrinsic cancer resistance, but also in patients that underwent several treatment courses and acquired cancer resistance (appearing after positive initial outcomes) [1]. Triple-negative breast cancers (TNBC), a breast cancer subtype with more aggressive biology, high rates of metastasis and potential relapse and mortality, stands out as having limited therapeutic options [2] and affects nearly 10–20% of all diagnosed breast cancers [3], mainly in younger (under forty years of age, [4]), African Americans [5], Hispanic women [6] and/or individuals with a BRCA1 gene mutation [4]. Platinum-(Pt)-based drugs are often used in neoadjuvant and salvage chemotherapy schemes to treat TNBC patients [7]. Nonetheless, severe adverse effects and toxicity, as well as cancer-acquired resistance, often occur during the time course of TNBC patients’ treatment [2,8,9], hampering their clinical success. To overcome drug resistance and therapy failure, substantial focus has been given to combinations of existing therapies, as well as to the development of novel drug entities with improved properties, selectivity and novel mechanisms of actions [10,11]. In this regard, metal complexes are at the forefront of research [12,13] owing to their particular properties regarding coordination environments/geometries and/or activation/inhibition of ligands under suitable conditions [13,14]. Such features might have an increased added pharmaceutical value, as demonstrated by conventional Pt(II) compounds (cisplatin, Figure 1; carboplatin or oxaliplatin) that have successfully been used as anticancer agents [15,16]. 

The therapeutic effectivity of these type of Pt(II) agents results, at least in part, from the formation of Pt-DNA adducts (through covalent binding to the bases), which induces DNA damage and triggers apoptosis [17]. As potential Pt(II) analogs, palladium(II) complexes have received particular attention owing to the similarities between these cations regarding both electronic configuration and coordination. Biogenic polyamines, namely spermidine (H_2_N(CH_2_)_4_NH(CH_2_)_3_NH_2_, Spd) and spermine (H_2_N(CH_2_)_3_NH(CH_2_)_4_NH(CH_2_)_3_NH_2_, Spm), have been identified as suitable polydentate ligands for both Pt^2+^ and Pd^2+^ ions, allowing the formation of stable chelates with appropriate kinetic properties [18], such as the trinuclear Pt(II) or Pd(II) chelates with spermidine (Pt_3_Spd_2_ and Pd_3_Spd_2_, Figure 1). In addition, Pd-based chelates are expected to display a higher tolerability and safety profiles as compared to cisplatin, carboplatin or oxaliplatin [12,19,20], a major advantage for proceeding with preclinical and clinical evaluations. Similar to conventional Pt(II)-based drugs (e.g., cisplatin), DNA interactions are the main mode of action of Pd(II) compounds. However, as for the analogous polynuclear Pt(II) chelates, these agents, containing more than one metal center, can bind to the DNA’s helix at more than one site and through long-range interstrand adducts, which prompts more severe and unrepairable damage compared to conventional, mononuclear Pt(II) drugs. Additionally, the impact of Pd(II) compounds has been reported to also occur on other targets apart from DNA, such as proteins [21], oxidative species [22,23] or even intracellular water [24].

Pt(II) and Pd(II) chelates spermine (Pt_2_Spm or Pd_2_Spm) have been reported to yield differentiated anticancer activity towards several human cancer cell lines, namely osteosarcoma [25], breast [26] and ovarian cancer [27]. Furthermore, in vivo studies allowed to determine the biodistribution and pharmacokinetic profile of Pd_2_Spm [20], as well as its selectivity/efficacy regarding TNBC cell growth inhibition in mice [19]. These results evidence the promising therapeutic potential of Pd(II) chelates with biogenic polyamines and foster further research on other similar complexes. The current study focuses on the Pd(II) and Pt(II) trinuclear chelates with spermidine (Pd_3_Spd_2_ and Pt_3_Spd_2_) and aims to evaluate its putative anticancer activity towards cisplatin-resistant TNBC cells (MDA-MB-231/R), as compared to cisplatin-sensitive TNBC (MDA-MB-231), by assessing the (i) antiproliferative activity, (ii) selectivity and (iii) ability to overcome cancer resistance. Furthermore, Pd_3_Spd_2_ activity will be compared with the effects elicited by (i) its Pt(II) analog, Pt_3_Spd_2_, (ii) cisplatin (platinum reference drug used in the clinical practice) and (iii) Pd_2_Spm (formerly investigated, with very promising results towards TNBC).

## 2. Materials and Methods

### 2.1. Reagents and Chemicals

Acetic acid glacial (99.7%), cisplatin (cis-dichlorodiammine platinum(II), 99.9%), (3-(4,5-dimethylthiazol-2-yl)-2,5-diphenyltetrazolium bromide (MTT), Dulbecco’s Modified Eagle’s Medium—high-glucose cell growth medium (DMEM-HG), 1:1 mixture of Dulbecco’s Modified Eagle’s Medium and Ham’s F12 cell growth medium (DMEM/F12 1:1), human epidermal growth factor (hEGF; recombinant, expressed in *E. coli*), cholera toxin from *Vibrio cholerae*, bovine insulin (10 mg/mL insulin in 25 mM HEPES, pH 8.2), hydrocortisone, dimethyl-sulphoxide (DMSO), phosphate-buffered saline (PBS), potassium tetrachloropalladate (II) (K_2_PdCl_4_, 98%), potassium tetrachloroplatinate (II) (K_2_PtCl_4_, 98%), sodium bicarbonate (NaHCO_3_, ≥99.0%), spermidine (*N*^1^-(3-Aminopropyl)butane-1,4-diamine, 99%), Sulforhodamine B (SRB), trypan blue (0.4% *w*/*v*) and trypsin-EDTA (1×), as well as solvents, inorganic salts and acids, were purchased from Sigma-Aldrich Chemical S.A. (Sintra, Portugal). Fetal bovine serum (FBS) and horse serum were acquired from Gibco-Life Technologies (Porto, Portugal). The DMSO concentration in the culture medium did not exceed 0.1% (*v*/*v*) and was considered as the control. 

### 2.2. Synthesis and Formulation of the Pd_3_Spd_2_, Pt_3_Spd_2_ and Pd_2_Spm

The Pd_3_Spd_2_ and Pt_3_Spd_2_ complexes were synthesized according to published procedures [28,29] optimized by the authors: 3 mmol of K_2_PdCl_4_ or K_2_PtCl_4_ was dissolved in a minimal amount of water, and 1.98 mmol of spermidine trihydrochloride aqueous solution was added dropwise under continuous stirring. After 15 min, the resulting orange precipitate was isolated by filtration and washed with water, ethanol and acetone and air-dried. The Pd_2_Spm complex was synthesized according to published procedures [30] optimized by the authors [31]. The composition and purity of the synthesized compounds were obtained by elemental analysis and vibrational spectroscopy (FTIR, Raman and inelastic neutron scattering), which were compared with the previously calculated vibrational profiles (by DFT methods) [31]. All stock solutions were freshly prepared by dissolving an appropriate quantity of drug in PBS containing 10% of DMSO and then sterile filtered.

### 2.3. Cell Cultures Development and Maintenance

The human TNBC MDA-MB-231 cell line (ATCC HTB-26) and the non-cancerous breast cell line MCF-12A (ATCC CRL-10782) were purchased from ATCC (Manassas, VA, USA). DMEM-HG cell growth medium supplemented with 10% (*v*/*v*) FBS was used to culture breast cancer cells. To establish a cisplatin-resistant cell strain, MDA-MB-231 cells were continuously treated with increasing concentrations of cisplatin (up to 2 µM) during a period of 6 months. When a consistent cell growth rate in the presence of cisplatin was achieved, these cells were named MDA-MB-231/R and stocked to assure the consistency of the phenotype for future use and experiments. All posterior experiments were performed within 10 passages to maintain the resistance while routinely growing the MDA-MB-231/R cell line in a cell culture medium without the addition of cisplatin. MCF-12A cells were cultured in DMEM/F12 medium supplemented with 100 ng/mL cholera toxin, 0.01 mg/mL bovine insulin, 20 ng/mL hEGF, 500 ng/mL hydrocortisone and 5% (*v*/*v*) horse serum. Cells were grown in monolayers in a sterile environment at 37 °C with a 5% CO_2_ humidified atmosphere. Under these conditions, the population doubling time was 25.5 ± 0.9 h and 30.6 ± 1.1 h for MDA-MB-231 and MDA-MB-231/R cells, respectively, and 20.6 ± 3.1 h for MCF-12A cells. The cell cultures were routinely screened for mycoplasma contamination, yielding negative results.

### 2.4. Cell Proliferation Evaluation

Cells were seeded in 96-well microplates at the cell density 1.5 × 10^4^ cells/cm^2^ (final volume 200 µL/well) and left 24 h to attach. Afterwards, the growth medium was replaced with the growth medium containing cisplatin (0.1–200 µM), Pd_3_Spd_2_ (0.3–200 µM), Pt_3_Spd_2_ (0.3–100 µM) and Pd_2_Spm (0.3–200 µM).

Cell proliferation was monitored with the direct image acquisition of cells on microplates at 0, 24, 48 and 72 h post-addition of the tested compounds using a LionheartFX automated microscope (BioTek, Winooski, VT, USA). Label-free kinetic 4× images were acquired and processed using Gen 5 Image Analysis software (BioTek, Winooski, VT, USA). Individual cells per image were identified and counted, and normalized cell growth (%) was calculated using the following formula:

$$C\left(t\right)=\left[\frac{\left(\mathrm{Number}\;\mathrm{of}\;\mathrm{Treated}\;\mathrm{Cells}\left(t\right)-\mathrm{Number}\;\mathrm{of}\;\mathrm{Treated}\;\mathrm{Cells}\left(0\right)\right)}{\left(\mathrm{Number}\;\mathrm{of}\;\mathrm{Untreated}\;\mathrm{Cells}\left(t\right)-\mathrm{Number}\;\mathrm{of}\;\mathrm{Untreated}\;\mathrm{Cells}\left(0\right)\right)}\right]\times 100$$

where C(t) is the percent of net cell growth over time, Number of Treated Cells(t) is the count of cells treated with drug at each time point, Number of Treated Cells(0) is the count cells treated with drug at time 0 h, Number of Untreated Cells(t) is the count of untreated (control) cells at each time point and Number of Untreated Cells(0) is the count of untreated (control) cells at time 0 h.

The Sulforhodamine B (SRB) [32] method was performed after each incubation period (24, 48 and 72 h). Briefly, the cells were washed with PBS and Mili-Q water and fixed with 1% (*v*/*v*) acetic acid in methanol overnight at −20 °C, followed by the addition of 100 µL SRB solution and incubation for 1h at 37 °C. The cells where then thoroughly washed with 1% acetic acid (*v*/*v*) and left to dry at room temperature. Then, a solubilization of the SRB dye with TRIS (10 mM, pH = 10), followed by the optical density measured at 540 nm, was carried out.

### 2.5. MTT Assay

The cells were harvested upon the addition of trypsin/EDTA solution, seeded in 96-well plates (1.5 × 10^4^ cells/cm^2^) and incubated at 37 °C. After allowing the cells to adhere for 24 h, the growth medium was replaced with the growth medium containing Pd_3_Spd_2_ (1–200 µM) or Pt_3_Spd_2_ (1–200 µM).

After each incubation period (24, 48 and 72 h), the cell viability was evaluated by the MTT [33] method. Briefly, a MTT solution (0.5 mg/mL in PBS) was added to each well after carefully removing the medium and allowed to incubate for 3 h at 37 °C; after which, the MTT was aspirated and, in order to dissolve the formazan crystals, 100 µL of DMSO were added. The optical density was measured at 570 nm.

### 2.6. Statistical Data Analysis

Data are expressed as the mean ± standard error of the mean (SEM). GraphPad Prism 7 Software (San Diego, CA, USA) was used, and the statistical analysis was performed using the two-tailed Student’s *t*-test. The half-maximal inhibitory concentration values (IC_50_) were calculated by interpolation from the nonlinear regression curves obtained from cell proliferation/cytotoxicity data using “log(inhibitor) versus the normalized response–variable slope equation”. A *p*-value lower than 0.05 was considered statistically significant.

## 3. Results

Both Pd_3_Spd_2_ and Pt_3_Spd_2_ are linear polyamine complexes comprising three metal centers coordinated to two polydentate spermidine ligands (each of them containing three N coordinating atoms), yielding highly stable chelates. This stabilization reduces the characteristic kinetic lability of the Pd(II) compounds. Pd_3_Spd_2_ and Pt_3_Spd_2_ can bind with high affinity to the nitrogen atoms of the DNA bases, mainly to the purines (the adduct with guanine being favored by intramolecular H-bonds). Their chemical features are responsible for an amphiphilic character and a high flexibility that enable an effective interaction with the DNA’s double helix at distinct sites, both intra- and interstrand. Additionally, the hydrophobic moiety of the complexes (carbon aliphatic chains) allows quite a strong interplay with the DNA’s backbone (pre-association), which favors the Pd–N- or Pt-N-binding process.

### 3.1. Impact of Platinum (Pt(II)) and Palladium (Pd(II)) Trinuclear Chelates with Spermidine on the Proliferation of Triple-Negative Breast Cancer 

**Figure 2:**
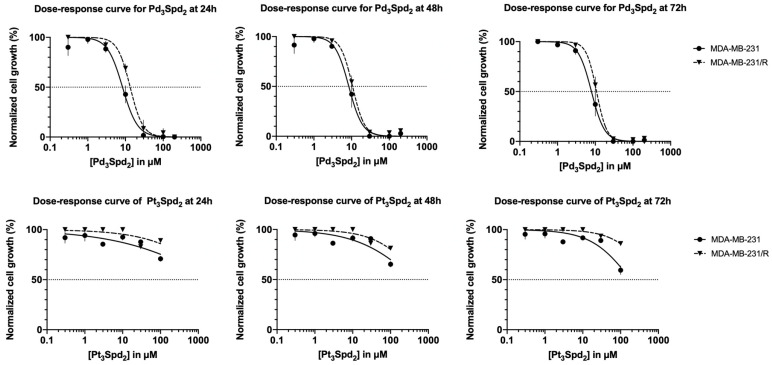
Impact of increasing concentrations of Pd_3_Spd_2_ or Pt_3_Spd_2_ on TNBC proliferation. Dose–response curves of Pd_3_Spd_2_ (**upper panel**) and Pt_3_Spd_2_ (**lower panel**) in breast cancer MDA-MB-231 and MDA-MB-231/R cells at 24, 48 and 72 h of incubation. Values are expressed as the mean ± SEM from 4 independent experiments (in triplicate) analyzed with nonlinear regression and the Student’s *t*-test. Data points with no visible error bars have errors smaller than the size of the symbol.

Firstly, the suppression of MDA-MB-231 cell proliferation induced by the palladium and platinum spermidine complexes was evaluated at 24, 48 and 72 h of incubation within a concentration range between 0.2 and 200 μM (Figure 2). 

Only the Pd(II) trinuclear chelates with spermidine Pd_3_Spd_2_ suppressed cell proliferation with concentrations above 1 μM, and the antiproliferative effect was concentration-dependent. Pt(II) trinuclear chelates with spermidine Pt_3_Spd_2_ induced a slight reduction of cell proliferation (up to 25%) but only with concentrations above 100 μM. In fact, for Pd_3_Spd_2_, the IC_50_ values ranged between 7.92 and 8.34 µM in MDA-MB-231 cells, depending on the incubation period, while the Pt_3_Spd_2_′s IC_50_ values were higher than 100 μM for all incubation periods (Table 1). Moreover, when comparing the effects of 100 μM of Pt_3_Spd_2_ with the same concentration of Pd_3_Spd_2_ in MDA-MB-231 cells, significantly lower cell growth was obtained in the presence of Pd_3_Spd_2_ for all incubations.

To determine a potential effect against cell resistance to cisplatin, the palladium and platinum spermidine complexes were also studied on MDA-MB-231 cells that had previously developed resistance to cisplatin (denoted as MDA-MB-231/R cells) compared to drug-naïve MDA-MB-231 cells (Figure 2); the complexes revealed that only Pd_3_Spd_2_ is able to suppress MDA-MB-231/R cell proliferation. When comparing the effects of 100 μM of Pd_3_Spd_2_ with the same concentration of Pt_3_Spd_2_ in MDA-MB-231/R cells, significantly lower cell growth was obtained in the presence of Pd_3_Spd_2_ for all incubations. When comparing IC_50_ of Pd_3_Spd_2_ obtained for MDA-MB-231/R at 24, 48 and 72 h with the IC_50_ values of Pd_3_Spd_2_ obtained for MDA-MB-231 cells, the resistance index was 1.4, 1.3 and 1.4 for 24, 48 and 72 h of incubation, respectively. These data were confirmed using other methodology to assess the cell proliferation (using the SRB method; see Supplemental Materials Figure S1).

**Figure 3:**
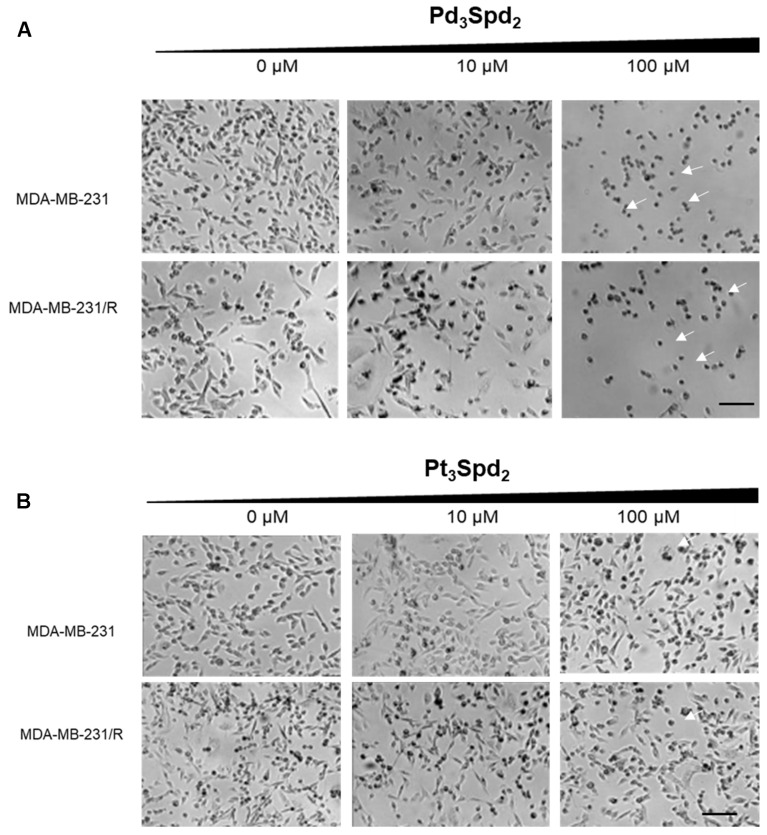
Representative photomicrographs with morphological features induced by increasing concentrations of Pd_3_Spd_2_ (**A**) or Pt_3_Spd_2_ (**B**) incubated for 48 h in MDA-MB-231 cells or MDA-MB-231/R cells. White arrows show cytoplasmic shrinkage and the rounding of cells. Representative images from 4 independent experiments obtained under an objective lens of a phase contrast of the LionheartFX microscope. Scale bar = 100 μm.

The microscopic observation of MDA-MB-231 and of MDA-MB-231/R cells treated with increasing concentrations of Pd_3_Spd_2_ or Pt_3_Spd_2_ for 48 h revealed various morphological changes but only for the cell lines treated with Pd_3_Spd_2_. In the contrast phase images, normal spindle-shaped phenotypes of MDA-MB-231 or MDA-MB2-231/R control cells contrasted with cells treated with Pd_3_Spd_2_ that exhibited cytoplasmic shrinkage and the rounding of cells (arrows), a low cell density and more floating cells in a dose-dependent manner (Figure 3).

**Figure 4:**
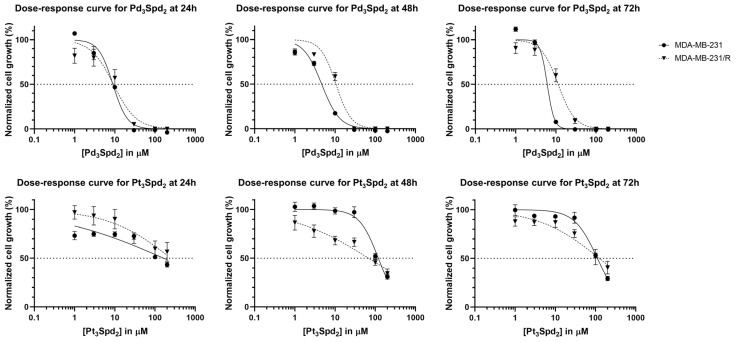
Impact of increasing concentrations of Pd_3_Spd_2_ or Pt_3_Spd_2_ on TNBC. Dose–response curves of Pd_3_Spd_2_ (**upper panel**) and Pt_3_Spd_2_ (**lower panel**) in breast cancer MDA-MB-231 and MDA-MB-231/R cells at 24, 48 and 72 h of incubation obtained through the MTT assay. Values are expressed as the mean ± SEM from 4 independent experiments (in triplicate) analyzed with nonlinear regression. Data points with no visible error bars have errors smaller than the size of the symbol.

The metabolic impact (in both MDA-MB-231 and MDA-MB-231/R) in the presence of Pd_3_Spd_2_ and Pt_3_Spd_2_ was evaluated measuring their ability to convert 3-[4,5-dimethylthiazol-2-yl]-2,5 diphenyl tetrazolium bromide into formazan, thus determining the mitochondrial activity (Figure 4) and cell viability.

A clear separation between the dose–response curves for MDA-MB-231 and MDA-MB-231/R was observed for both Pd_3_Spd_2_ and Pt_3_Spd_2_ at the 24, 48 and 72 h incubation times. For Pd_3_Spd_2_, a slight overlap between both cell lines was observed at 24 h. Table 2 comprises the IC_50_ values obtained for Pd_3_Spd_2_ and Pt_3_Spd_2_ for the 24, 48 and 72 h incubation periods. The values obtained for Pd_3_Spd_2_ were lower at all time points when compared to Pt_3_Spd_2_, demonstrating that both the Pd- and Pt-trinuclear chelates impact the mitochondrial activity but with different potencies. The resistant cells (MDA-MB-231/R) were found to be less sensitive to both Pt(II)/Pd(II) compounds when compared to the corresponding sensitive cells (MDA-MB-231).

### 3.2. Pd(II) Trinuclear Chelates with Spermidine Anticancer Potential

**Figure 5:**
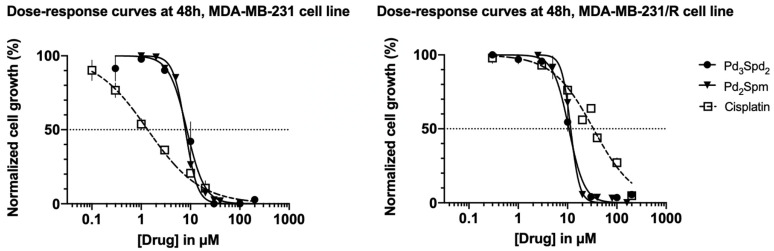
Impact of increasing concentrations of Pd_3_Spd_2_, cisplatin or Pd_2_Spm on TNBC sensitive and resistant to cisplatin incubated for 48 h. Dose–response curves in breast cancer MDA-MB-231 cells (sensitive to cisplatin, (**left panel**) line) and MDA-MB-231/R cells (resistant to cisplatin, (**right panel**)) at 48 h of incubation. Values are expressed as the mean ± SEM from 4 independent experiments (in triplicate) analyzed with nonlinear regression and the Student’s *t*-test. Data points with no visible error bars have errors smaller than the size of the symbol.

The ability of Pd(II) trinuclear chelates with spermidine Pd_3_Spd_2_ to suppress cell proliferation in MDA-MB-231 cells or MDA-MB-231/R cells was compared to the effects elicited by cisplatin ([34], a platinum reference drug used in clinical practice), as well as of Pd_2_Spm ([12], used as a referenced Pd compound with already described anticancer effects in several cell lines: oral squamous carcinoma [35] and osteosarcoma [25] and TNBC [19], as shown in Figure 5).

In sensitive cells (MDA-MB-231 cells; Figure 5, left panel), the two palladium complexes share a similar profile (*p* > 0.05) in inducing the suppression of cell proliferation, i.e., shown to have an equipotent effect: Pd_3_Spd_2_—IC_50_ (48 h) is 8.44 μM, and Pd_2_Spm—IC_50_ (48 h) is 7.90 μM. Maximum suppression is achieved with a concentration of 30 μM for both Pd_2_Spm and Pd_3_Spd_2_; *p* < 0.05. Cisplatin has a lower IC_50_ (48 h), 1 μM, when compared to the values obtained for the palladium complexes (*p* < 0.01), and the maximum suppression of cell proliferation is occurring with a concentration of only 30 μM. 

In cells resistant to cisplatin (MDA-MB-231/R cells; Figure 5, right panel), the maximum suppression of cell proliferation with cisplatin is only achieved with a much higher concentration, 200 μM (*p* < 0.001, compared to palladium complexes), and its IC_50_ (48 h) is 32.4 μM. The palladium complexes’ profiles regarding the cell proliferation of MDA-MB-231/R (resistant cells) are slightly shifted (*p* > 0.05) to the right compared to the ones obtained for MDA-MB-231 (sensitive cells), which is demonstrated by the Pd_3_Spd_2_ IC_50_ (48 h): 8.44 μM versus 10.63 μM (MDA-MB-231 versus MDA-MB-231/R) and Pd_2_Spm IC_50_ (48 h): 7.90 μM versus 11.6 μM (MDA-MB-231 versus MDA-MB-231/R). Despite this, the maximum suppression of cell proliferation in MDA-MB-231/R cells still occurs with a concentration of 30 μM (*p* < 0.05) for both Pd_2_Spm and Pd_3_Spd_2_.

Pd_3_Spd_2_ demonstrated a promising antineoplastic activity, which is lower than the cisplatin-mediated effects in sensitive cells but able to surpass its resistance. It further indicates that Pd_3_Spd_2_ promotes an impact on cancer cells similar to Pd_2_Spm in both sensitive and resistant cells.

### 3.3. Pd(II) Trinuclear Chelates with Spermidine Selectivity 

**Figure 6:**
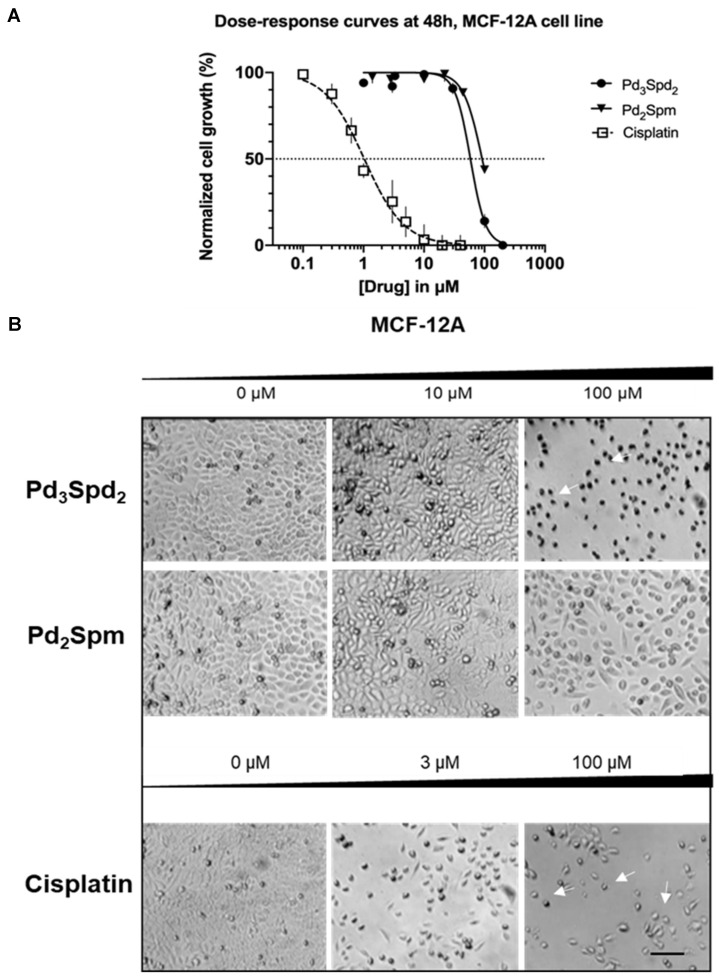
Impact of increasing concentrations of Pd_3_Spd_2_, Pd_2_Spm or cisplatin on breast healthy cells (MCF-12A) incubated with the drug for 48 h. (**A**) Dose–response curves for cell proliferation. Values are expressed as the mean ± SEM from 4 independent experiments (in triplicate) analyzed with nonlinear regression and the Student’s *t*-test. Data points with no visible error bars have errors smaller than the size of the symbol. (**B**) Representative photomicrographs showing morphological changes. White arrows show cytoplasmic shrinkage and the rounding of cells. Representative images from 4 independent experiments obtained under an objective lens of a phase contrast of the LionheartFX microscope. Scale bar = 100 μm.

The Pd(II) trinuclear chelate with spermidine Pd_3_Spd_2_ was also tested in healthy breast cells (MCF-12A), and the results obtained were compared to the effects elicited by either Pd_2_Spm or cisplatin incubated for 24 ([19]), 48 (Figure 6A) and 72 h ([19]). Cisplatin, incubated for 48 h, was revealed to suppress MCF-12A cell proliferation in a concentration-dependent manner with a profile very similar to that obtained for MDA-MB-231-sensitive cells (IC_50_ is 1 μM for both cells lines with a selectivity index of 1). Pd_3_Spd_2_ was also able to suppress MCF-12A cell proliferation but required higher concentrations (above 200 μM). In contrast, Pd_2_Spm failed to promote such an effect in the range of concentrations tested: Pd_2_Spm can modify healthy cell proliferation but only with concentrations above 40 μM, inducing suppression up to 60% (for 100 μM). In fact, regarding Pd_3_Spd_2_, the IC_50_ (48 h) for MCF-12A was 53 μM, thus attaining a selectivity index of 6.28 or 4.99 for MDA-MB-231 or MDA-MB-231/R, respectively, at 48 h. For other incubation periods (24 h or 72 h), the dose–response curve profile of Pd_3_Spd_2_ is similar, and the selectivity indexes are depicted in Table 3.

The morphological features of MCF-12A were maintained after 48 h of incubation of both palladium complexes, but changes were evident in the presence of cisplatin (Figure 6B).

For the higher concentration tested, various morphological changes were observed for cells treated with Pd_3_Spd_2_ and cisplatin with cell cytoplasmic shrinkage and the rounding of cells (arrows), low cell density and more floating cells (Figure 6B).

## 4. Discussion

This study reveals, for the first time, a promising anticancer potential of a trinuclear Pd(II) chelate with spermidine (Pd_3_Spd_2_) towards human TNBC. The current results clearly evidenced that Pd_3_Spd_2_ seems to be the most effective agent regarding the suppression of TNBC cell proliferation (Figure 2) when compared to the analogous Pt(II) compound (Pt_3_Spd_2_), which had evidenced only a moderate impact on this cancer type. Accordingly, data previously reported for Pt_2_Spm and Pd_2_Spm showed a significantly lower antineoplastic effect for the Pt(II) complex towards several human cancer cells, namely ovarian cancer [27], osteosarcoma [25] and TNBC [36]. 

Pd_3_Spd_2_ elicited a deleterious effect in mitochondrial function (measured through the MTT assay), which is absent in Pt_2_Spm-treated cells. As previously reported, the initial rate of formazan formation may serve as an indicator of complex I activity and the pyruvate transport rate [37], indicating a disruption of mitochondrial functional activity, conditioning cell metabolism and, therefore, cell viability. Previous metabolomic studies of osteosarcoma in mice in the presence of Pd_2_Spm have highlighted its impact on osteoblastic amino acid metabolism [38], as well as numerous changes in specific amino acids, nucleotides and derivatives; membrane precursors (choline and phosphoethanolamine); dimethylamine; fumarate and guanidine acetate [23]. These data prompt the need for similar metabolomic studies in order to clarify the impact of the currently investigated Pd_3_Spd_2_ on TNBC, which may help to understand why different palladium-based chelates (namely, dinuclear with Spm and trinuclear with Spd) exhibit comparable antineoplastic activities towards TNBC, as reported in the present study. Indeed, the equipotent effect evidenced by these Pd(II) di- or trinuclear polyamine chelates (Pd_3_Spd_2_ and Pd_2_Spm) highlight that their anticancer capacity cannot be directly related to the biogenic polyamine used as a ligand (either spermine or spermidine), nor to the distinct DNA adducts formed upon drug interactions, but, rather, to a shared target (DNA) and mechanism of action that trigger the observed metabolic changes.

Similar to Pd_2_Spm, palladium’s anticancer activity is mainly due to an unconventional interaction with the DNA at more than one site in the double helix, yielding long-range interstrand adducts, which triggers a cellular damage usually more severe and unrepairable than that ascribed to cisplatin [18], as previously described for osteosarcoma [25]. By contrast, there have also been studies reporting a higher anticancer effect for cisplatin relative to Pd_2_Spm towards prostatic cancer [39], in line with the currently obtained lower antineoplastic effects of palladium chelates compared to cisplatin against MDA-MB-231 cells. Another example is the activity described for the palladium compound Pd(bpy)(ONO_2_)_2_, reported to act as an effective antitumor agent against sarcoma [40] while it is inactive towards leukemic P388 cells [41]. These results suggest that the compounds’ efficacy (either Pt- or Pd-based) also depends on the cancer type. Nevertheless, further studies are required to identify the mechanism(s) of action and type of cell death triggered by these compounds, considering that some novel metallodrugs seem to promote their antineoplastic effects through apoptosis [42], necroptosis [43], pyroptosis [44], ferroptosis [45] or autophagic cell death [46].

Particularly relevant is the ability of Pd_3_Spd_2_ to overcome cancer resistance to cisplatin. This constitutes a highly promising feature currently described for the first time for trinuclear Pd(II) chelates and previously observed for the dinuclear Pd_2_Spm complex [19]. Such activity is of major relevance, since cancer resistance to drugs constitutes, at present, the main limiting factor to achieving a cure for cancer patients [10,11]. The dose–response profiles for Pd_3_Spd_2_ and Pd_2_Spm overlap in MDA-MB-231/R cells, revealing a similar efficacy and potency towards these resistant TNBC cells: IC_50_ values equal to 10.63 μM versus 11.6 μM for Pd_3_Spd_2_ and Pd_2_Spm, respectively. Hence, both Pd-derived compounds appear to be suitable alternatives as chemotherapeutic agents against drug-resistant cancers, responding to the challenge posed by Vasan and coworkers (2019) to develop novel drug entities able to overcome drug resistance and therapy failure [10].

Cancer therapy failure may also be associated with factors other than drug resistance. In this respect, scientific evidence has highlighted that the severity of adverse effects, due to a low selectivity of drugs, has a great deleterious impact on patients’ quality of life and deeply influences the dosing regimen, leading to a poor chemotherapy response [47,48]. The cisplatin treatment for 48 h currently showed a selectivity index of 1, i.e., both TNBC and healthy mammary cells presented an equivalent response to cisplatin (cisplatin is as deleterious to breast cancer as to healthy cells). Ideally, a drug should kill the cancer cells but should not affect the healthy cells [49]; thus, the selectivity index should be the highest possible [50]. For the currently investigated complex Pd_3_Spd_2_, the suppression of proliferation in healthy cells requires a much higher dosage than that needed to promote an equivalent effect on MDA-MB-231 cells, for example, IC_50_ at 48 h for healthy cells is 53.00 μM, whereas, for MDA-MB-231 cells, it is 8.44 μM. The selectivity index exhibited by Pd_3_Spd_2_ varies (depending on the time of incubation) between 6.28 and 7.94 for sensitive TNBC and 4.59 and 4.99 for resistant TNBC. In turn, Pd_2_Spm presently showed a clear selectivity towards TNBC cells (IC_50_ equal to 7.9 μM for TNBC versus ca. 90 μM for MCF-12A cells, at 48 h), confirming the data previously reported [19].

## 5. Conclusions

The trinuclear chelates Pd_3_Spd_2_ and Pt_3_Spd_2_ were investigated in the current study, and the results obtained unveiled (i) an improved antiproliferative activity (surpassing resistance to cisplatin), (ii) anticancer selectivity and (iii) equipotency towards TNBC (both in cisplatin-sensitive and cisplatin-resistant cells). This study also revealed that Pd_3_Spd_2_ activity greatly exceeds that displayed by their respective Pt analog (Pt_3_Spd_2_), similarly to the results gathered for the Pd_2_Spm/Pt_2_Spm chelates.

In addition, Pd_2_Spm evidenced that the antiproliferative activity of trinuclear Pd(II) was comparable to the dinuclear analog (Pd_2_Spm). Further studies are needed to track the antineoplastic activities and to explore the targets and mechanism(s) of action triggered by these types of Pd(II)-based compounds. Overall, however, we can consider, based on the features gathered so far, that Pd_3_Spd_2_ can be included in the role of new compounds that fulfill the demands required by promising anticancer agents.

## Figures and Tables

**Table 1:** Half-maximal inhibitory concentration (IC_50_) of Pd_3_Spd_2_ or Pt_3_Spd_2_ in MDA-MB-231 and MDA-MB-231/R cells at 24, 48 and 72 h of incubation regarding the suppression of cell proliferation.

Drug	Incubation Time	MDA-MB-231 IC_50_ (µM)	MDA-MB-231/R IC_50_ (µM)	Resistance Index
Pd_3_Spd_2_	24 h	8.35	13.34	1.6
	48 h	8.44	10.63	1.3
	72 h	7.92	10.82	1.4
Pt_3_Spd_2_	24 h	>100	>100	N/A
	48 h	>100	>100	N/A
	72 h	>100	>100	N/A

IC_50_ values (cell proliferation assay) are expressed in µM, *n* = 4. Resistance index was calculated as a ratio: IC_50_ of the drug-resistant cell line (MDA-MB-231/R) divided by the IC_50_ value obtained in the drug-sensitive cell line (MDA-MB-231) at respective time points.

**Table 2:** Half-maximal inhibitory concentration (IC_50_) of Pd_3_Spd_2_ or Pt_3_Spd_2_ against MDA-MB-231 and MDA-MB-231/R cells at 24, 48 and 72 h of incubation regarding cell viability measurements.

Drug	Incubation Time	MDA-MB-231 IC_50_ (µM)	MDA-MB-231/R IC_50_ (µM)
Pd_3_Spd_2_	24 h	8.99	9.24
	48 h	4.65	10.57
	72 h	6.12	11.37
Pt_3_Spd_2_	24 h	>100	>100
	48 h	>100	67.86
	72 h	>100	>100

IC_50_ values (MTT assay) are expressed in µM, *n* = 4. Resistance index was calculated as a ratio: IC_50_ of the drug-resistant cell line (MDA-MB-231/R) divided by the IC_50_ value obtained in drug-sensitive cell line (MDA-MB-231) at the respective time points.

**Table 3:** Half-maximal inhibitory concentration (IC_50_) of Pd_3_Spd_2_ against MCF-12A cells at 24, 48 and 72 h of incubation regarding the suppression of cell proliferation.

Incubation Time	IC_50_ (µM)	Selectivity Index for MDA-MB-231	Selectivity Index for MDA-MB-231/R
24 h	66.30	7.94	4.95
48 h	53.00	6.28	4.99
72 h	49.70	6.28	4.59

IC_50_ values (cell proliferation assay) are expressed in µM, *n* = 4. Selectivity index was calculated as a ratio: IC_50_ of the healthy cells (MCF-12A) divided by the IC_50_ value obtained either for the drug-sensitive cell line (MDA-MB-231) or for the drug-resistant cell line (MDA-MB-231/R) at the respective time points.

## Data Availability

The data presented in this study are available on request.

## Supplementary Materials

The following supporting information can be downloaded at: https://www.mdpi.com/article/10.3390/pharmaceutics15041205/s1: Figure S1: Impact of increasing concentrations of Pd_3_Spd_2_ or Pt_3_Spd_2_ on TNBC. Dose–response curves of Pd_3_Spd_2_ (upper panel) and Pt_3_Spd_2_ (lower panel) in breast cancer MDA-MB-231 and MDA-MB-231/R cells at 24, 48 and 72 h of incubation obtained through the SRB assay. Values are expressed as the mean ± SEM from 4 independent experiments (in triplicate). Data points with no visible error bars have errors smaller than the size of the symbol.
