# Supplementary material for: Pd(II) and Pt(II) Trinuclear Chelates with Spermidine: Selective Anticancer Activity towards TNBC-Sensitive and -Resistant to Cisplatin
Source: Pharmaceutics. 2023 Apr 10;15(4):1205. doi: 10.3390/pharmaceutics15041205 (PMC10145437; doi:10.3390/pharmaceutics15041205)
Supplement: Supplementary file 1 [file pharmaceutics-15-01205-s001.zip › pharmaceutics-2283480-supplementary.pdf]

Pd(II) and Pt(II) trinuclear chelates with spermidine: selective anticancer activity towards TNBC-sensitive and -resistant to cisplatin

Martin Vojtek[1, †], Clara B. Martins[2, †], Raquel Ramos[1], Sara Gomes Duarte[1], Isabel M. P. L. V. O. Ferreira[3], Ana L. M. Batista de Carvalho[2], M. Paula M. Marques[2,4] and Carmen Diniz[1]

[1] LAQV/REQUIMTE, Laboratory of Pharmacology, Department of Drug Sciences, Faculty of Pharmacy, University of Porto, 4050-313 Porto, Portugal

[2] Molecular Physical-Chemistry R&D Unit, Department of Chemistry, University of Coimbra, 3004-535 Coimbra, Portugal

[3] LAQV/REQUIMTE, Laboratory of Bromatology and Hydrology, Department of Chemical Sciences, Faculty of Pharmacy, University of Porto, 4050-313 Porto, Portugal;

[4] Department of Life Sciences, Faculty of Science and Technology, University of Coimbra, 3000-456 Coimbra, Portugal.

Supplementary Material

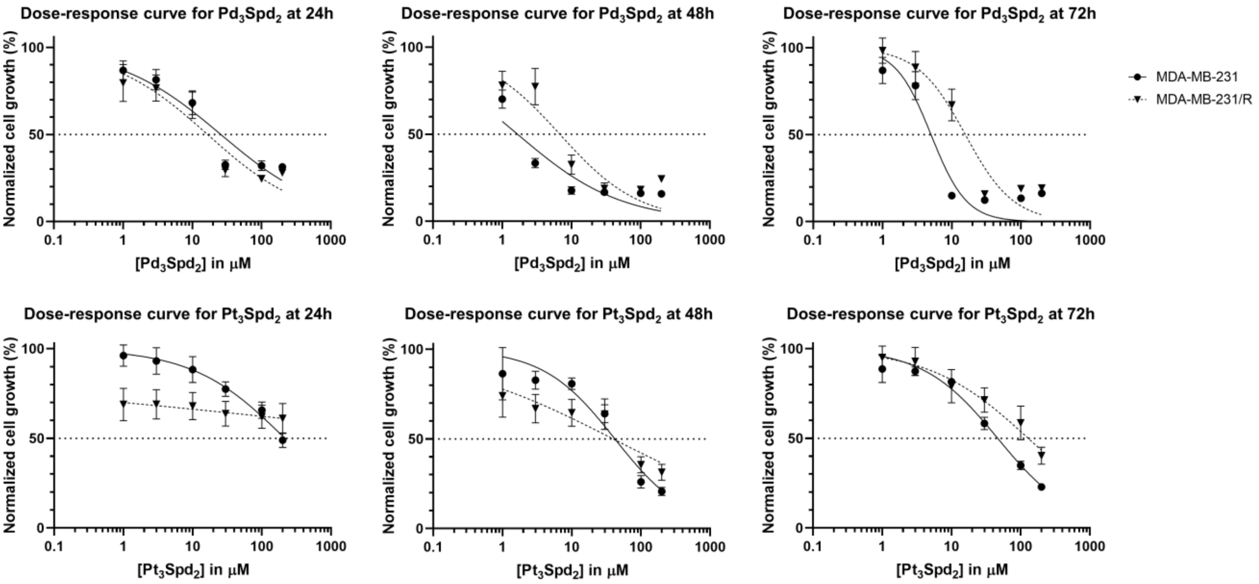

Figure S1. Impact of increasing concentrations of Pd_3Spd_2 or Pt_3Spd_2 on TNBC. Dose–response curves of Pd_3Spd_2 (upper panel) and Pt_3Spd_2 (lower panel) in breast cancer MDA-MB-231 and MDA-MB-231/R cell at 24, 48 and 72 h of incubation obtained through the SRB assay. Values are expressed as mean ± SEM from 4 independent experiments (in triplicate). Data points with no visible error bars have errors smaller than the size of the symbol.
